# Pre-Omicron Vaccine Breakthrough Infection Induces Superior Cross-Neutralization against SARS-CoV-2 Omicron BA.1 Compared to Infection Alone

**DOI:** 10.3390/ijms23147675

**Published:** 2022-07-12

**Authors:** Eveline Santos da Silva, Michel Kohnen, Georges Gilson, Therese Staub, Victor Arendt, Christiane Hilger, Jean-Yves Servais, Emilie Charpentier, Olivia Domingues, Chantal J. Snoeck, Markus Ollert, Carole Seguin-Devaux, Danielle Perez-Bercoff

**Affiliations:** 1 HIV Clinical and Translational Research Unit, Department of Infection and Immunity, Luxembourg Institute of Health, 29 rue Henri Koch, L-4354 Esch-sur-Alzette, Luxembourg; eveline.santosdasilva@lih.lu (E.S.d.S.); jean-yves.servais@lih.lu (J.-Y.S.); carole.devaux@lih.lu (C.S.-D.); 2 Centre Hospitalier de Luxembourg, 4 rue Ernest Barblé, L-1210 Luxembourg, Luxembourg; kohnen.michel@chl.lu (M.K.); gilson1@pt.lu (G.G.); staub.therese@chl.lu (T.S.); arendt.vic@chl.lu (V.A.); 3 Molecular and Translational Allergology, Department of Infection and Immunity, Luxembourg Institute of Health, 29 rue Henri Koch, L-4354 Esch-sur-Alzette, Luxembourg; christiane.hilger@lih.lu; 4 Clinical and Applied Virology, Department of Infection and Immunity, Luxembourg Institute of Health, 29 rue Henri Koch, L-4354 Esch-sur-Alzette, Luxembourg; emilie.charpentier@lih.lu (E.C.); chantal.snoeck@lih.lu (C.J.S.); 5 Allergy and Clinical Immunology, Department of Infection and Immunity, Luxembourg Institute of Health, 29 rue Henri Koch, L-4354 Esch-sur-Alzette, Luxembourg; olivia.domingues@lih.lu (O.D.); markus.ollert@lih.lu (M.O.)

**Keywords:** SARS-CoV-2, variants of concern (VOCs), neutralizing antibodies, convalescent sera, breakthrough infection, Delta, Omicron BA.1

## Abstract

SARS-CoV-2 variants raise concern because of their high transmissibility and their ability to evade neutralizing antibodies elicited by prior infection or by vaccination. Here, we compared the neutralizing abilities of sera from 70 unvaccinated COVID-19 patients infected before the emergence of variants of concern (VOCs) and of 16 vaccine breakthrough infection (BTI) cases infected with Gamma or Delta against the ancestral B.1 strain, the Gamma, Delta and Omicron BA.1 VOCs using live virus. We further determined antibody levels against the Nucleocapsid (N) and full Spike proteins, the receptor-binding domain (RBD) and the N-terminal domain (NTD) of the Spike protein. Convalescent sera featured considerable variability in the neutralization of B.1 and in the cross-neutralization of different strains. Their neutralizing capacity moderately correlated with antibody levels against the Spike protein and the RBD. All but one convalescent serum failed to neutralize Omicron BA.1. Overall, convalescent sera from patients with moderate disease had higher antibody levels and displayed a higher neutralizing ability against all strains than patients with mild or severe forms of the disease. The sera from BTI cases fell into one of two categories: half the sera had a high neutralizing activity against the ancestral B.1 strain as well as against the infecting strain, while the other half had no or a very low neutralizing activity against all strains. Although antibody levels against the spike protein and the RBD were lower in BTI sera than in unvaccinated convalescent sera, most neutralizing sera also retained partial neutralizing activity against Omicron BA.1, suggestive of a better cross-neutralization and higher affinity of vaccine-elicited antibodies over virus-induced antibodies. Accordingly, the IC50: antibody level ratios were comparable for BTI and convalescent sera, but remained lower in the neutralizing convalescent sera from patients with moderate disease than in BTI sera. The neutralizing activity of BTI sera was strongly correlated with antibodies against the Spike protein and the RBD. Together, these findings highlight qualitative differences in antibody responses elicited by infection in vaccinated and unvaccinated individuals. They further indicate that breakthrough infection with a pre-Omicron variant boosts immunity and induces cross-neutralizing antibodies against different strains, including Omicron BA.1.

## 1. Introduction

Two years into the COVID-19 pandemic which originated in Wuhan, China, in December 2019, SARS-CoV-2 has officially infected over 551 million individuals and claimed more than 6.34 million lives as of 3 July 2022. SARS-CoV-2 variants continuously mold the pandemic landscape. Variants that spread faster or elude immunity conferred by prior infection or by vaccines readily outcompete established strains. The first evolution of the Wuhan strain (B.1) emerged between mid-February and mid-March 2020 [1,2,3]. It harbors the D614G mutation in the Spike protein (S-D614G), which favors viral infectivity and transmission by positioning the Spike protein in an “up” position prone to bind ACE-2 [4]. Numerous other lineages have since stemmed from B.1, including the main variants of concern (VOCs) Alpha (B.1.1.7), Beta (B.1.351), Gamma (P.1), Delta (B.1.617.2) and Omicron (B.1.1.529). Aside from an increased transmission, infection with the Alpha and Delta variants is associated with higher disease severity and fatality rates [5,6,7,8,9,10,11,12,13,14], including in children and adolescents [15,16]. The recent Omicron variants BA.1 and BA.2, which were first identified in sub-Saharan Africa in November 2021, have supplanted Delta within a few weeks, causing two twin peaks in most countries [17]. Their infectivity and transmissibility are far higher than Delta and yet more transmissible sublineages are now blossoming. These variants are in turn being outgrown by BA.2 sublineages (notably BA.2.12.1) and by BA.4 and BA.5 [18,19]. The large number of deletions and mutations (>60 across the genome, including 37 in the viral Spike protein and R203K in the Nucleocapsid N), improve its affinity for the viral receptor ACE2 on target cells [20,21,22,23] and thereby its transmissibility, accelerate viral assembly and enable immune evasion [24,25,26,27,28]. Omicron variants cause less severe forms of COVID-19 (~60% lower risk of hospitalization or death compared to Delta) [20,29,30,31,32], reflecting the combined effects of intrinsic lower viral pathogenicity and prior immunity [14,33,34,35,36].

Encapsulated mRNA-based vaccines BNT162b2 (Pfizer-BioNTech) and mRNA-1273 (Moderna) as well as adenovector-based vaccines ChAdOx1 (Astra Zeneca, AZ) and Ad26.Cov2.S (Janssen) confer effective protection against severe forms of COVID-19 [10,37,38,39,40,41]. Protection against infection and transmission is less striking, particularly for the two latest VOCs Delta and Omicron [10,39,40,41,42,43,44,45,46,47,48,49,50], as testified by increasing numbers of reinfections and breakthrough infections [25,36,38,51,52,53,54,55,56,57,58]. The waning of immune responses and the emergence of variants with mutations in the Spike protein [25,26,27,37,38,39,40,43,48,49,51,52,59,60,61,62,63,64,65,66,67,68,69,70,71] are the main reasons of vaccine escape and reinfections. 

Convalescent patient sera and sera from individuals vaccinated with mRNA vaccines BNT162b or mRNA-1273 have lower in vitro neutralizing activity against VOCs than against B.1. For Alpha and Delta, the drop in neutralizing activity is modest (2–3-fold) [48,72,73,74,75,76,77], while it ranges from ~10-fold to full escape for Beta and Gamma [48,72,73,74,75,76,77,78]. In agreement with in vitro tests, the protection conferred by the BNT162b2 and the AZ ChAdOx1 vaccines against the Alpha variant is only moderately decreased [37,60,62,64]. Protection against the Delta variant dropped slightly, from >95% to 58–83% for BNT162b2 and from 70 to 60% for AZ ChAdOx1 [38,51,52,53,55,57,79]. Heterologous vaccination (AZ ChAdOx1 followed by an mRNA vaccine) shows the highest protection [57,80]. The cases of SARS-CoV-2 infection despite complete vaccination (hereafter referred to as breakthrough infection, BTI) have become very frequent since the emergence of the Omicron variants [25,45,46,47,56,57]. Accordingly, in vitro neutralization assays highlight a poor cross-neutralization of Omicron by sera from vaccinees [19,24,25,26,27,28,48,68,81,82]. Although booster vaccine doses restore neutralizing antibody (NAb) responses against Delta and partially against Omicron, they do not decrease viral load or transmission [27,28,44,46,48,57,80,81,82,83,84,85,86,87,88,89,90].

The protection against Omicron conferred by infection with other VOCs is still controversial. Some authors report that reinfection rates with Omicron are far superior to reinfection rates with Beta and Delta [91], and that NAbs elicited by pre-Omicron VOCs or by variant-specific vaccines are less effective against Omicron [36,48,68,80,92]. Others report that breakthrough infection with pre-Omicron VOCs elicits cross-reactive antibodies with the ability to neutralize Omicron [49,93,94]. Converging reports indicate that an Omicron BA.1 breakthrough infection elicits antibodies with neutralizing activity against Omicron BA.1 and pre-Omicron VOCs [36,48,92,95,96] but poorly cross-neutralizing other Omicron sublineages [97]. In unvaccinated individuals, antibodies elicited by an Omicron BA.1 infection neutralize BA.1 but not other VOCs [49,92,95]. The contribution of vaccine- or infection-induced immunity to the lower pathogenicity of Omicron and the long-term impact of infection by Omicron are difficult to set apart [14,33,35,36,92]. Nevertheless, the inverse correlation between antibody levels at the time of infection and viral load [36,98], together with the high rate of hospital admissions recorded in Hong-Kong, where vaccine coverage is low, suggest that pre-existing immunity plays a major role in protection. The widespread dissemination of Omicron calls for a better understanding of its sensitivity to neutralization by antibodies induced by vaccination and prior infection.

Because of its spectacular infectivity, the Omicron wave caused a substantial number of breakthrough infections and reinfections, and in some cases, even coinfections. These are a fertile ground for the surge of recombinants, which further complexify the epidemic landscape. Understanding the role of pre-existing immunity in this setting is thus fundamental. In this study, we compared the ability of convalescent sera from 70 unvaccinated patients infected with the ancestral pre-VOC B.1 strain and from 16 BTI cases infected with Gamma (2 patients) or Delta (14 patients) to neutralize Omicron BA.1, using replicating viral strains isolated from patients in Luxembourg. Pre-VOC convalescent sera showed substantial heterogeneity in their cross-neutralizing ability against pre-Omicron VOCs and generally failed to neutralize Omicron BA.1. Patients with moderate disease had overall higher neutralizing levels against all strains than patients with mild/asymptomatic disease or with severe or critical disease. In contrast, half the sera from BTI patients had a high neutralizing ability against all tested strains, while half had no or low NAbs against all strains. Neutralizing BTI sera retained some ability to neutralize Omicron, in line with previous reports [36,48,92,95,96]. Importantly, BTI sera had lower antibody levels than unvaccinated convalescent sera, suggesting higher affinity conferred by vaccination and hybrid immunity. 

## 2. Results

### 2.1. Cross-Neutralization of B.1, Gamma, Delta and OmicronBA.1 by Pre-VOC Convalescent Sera

#### 2.1.1. Cross-Neutralization of Convalescent Sera

**Figure 1:**
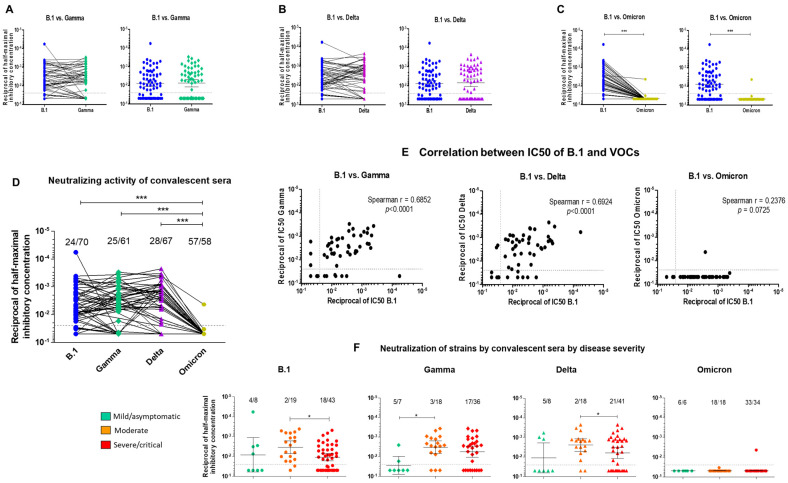
Neutralizing activity of pre-VOC unvaccinated SARS-CoV-2-infected convalescent sera against ancestral and VOC strains. (**A**–**C**). Pairwise comparison of half-maximal inhibitory concentrations (IC50) of B.1 (blue in each panel) with Gamma (green) (**A**), Delta (purple) (**B**) and Omicron (gold) (**C**). Serial two-fold dilutions (starting 1:40, grey dotted line on graph) of heat-inactivated convalescent sera were incubated for 1 h with 100 TCID50 of virus in viral growth medium. Vero-E6 cells (10^4^ cells/ well) were infected with the virus/serum mixture for 72 h at 37 °C. Virus-induced cytophathic effect (CPE) was measured using the tetrazolium salt WST-8, which is transformed into a red substrate by live cells. Optical density at 570 nm was measured to monitor live cells (uninfected cells). All infections were performed in triplicate wells. Percent survival was calculated relative to uninfected cells (100% survival) and the half-maximal inhibitory concentration for serum (IC50) was determined by inferring the 4-parameter nonlinear regression curve fit (GraphPad Prism v5). The top (100% survival) and bottom (no serum) values were unconstrained. (**D**). Comparison of IC50 for all strains. The infecting strain is indicated on the x-axis with the following color codes: blue = B.1, green = Gamma, purple = Delta, gold = Omicron BA.1. The proportion of non-neutralizing sera is indicated above each data set. (**E**). Correlation between the IC50 of sera against B.1 (x-axis in each panel) and VOCs (y-axis in each panel). The Spearman correlation coefficient (r) is indicated in each panel (**F**). IC50 of convalescent sera against B.1 and VOCs for patients with mild/asymptomatic (green), moderate (orange) or severe/critical (red) forms of COVID-19. The infecting strain is indicated above each panel. For all analyses, differences between groups were compared using a Mann–Whitney U-test for comparison between two groups and a Kruskal–Wallis followed by a Dunn’s multiple comparison post-hoc test when three or more groups were compared. *p*-values < 0.05 were considered significant. *: *p* < 0.05; **: *p* < 0.01; ***: *p* < 0.001.

First, we measured the neutralizing ability of sera from 70 patients infected early in the pandemic with the ancestral pre-VOC D614G (B.1) strain. The sera were collected during acute infection (median 14 days, interquartile range (IQR) 9–20) and most were from patients with moderate or severe/critical COVID-19. The half-maximal inhibitory concentrations (IC50) of convalescent sera against B.1, Gamma and Delta spanned a broad neutralization range and had similar geometric means (*p* > 0.05): IC50 B.1 = 0.007783, IC50 Gamma = 0.007653 and IC50 Delta = 0.007137 (Figure 1A,B and Supplementary Figure S1A–C). One third (24/70, 34.3%) of the convalescent sera failed to neutralize B.1, and a slightly but significantly higher proportion failed to neutralize Gamma (25/61, 41%, *p* < 0.01) and Delta (29/67, 43.3%, *p* < 0.01) at the highest serum concentration used (1:40). In contrast, all but one serum (57/58, 98.2%, *p* < 0.0001) failed to neutralize Omicron at the 1:40 serum dilution and the geometric mean IC50 was significantly higher (IC50 = 0.04676, *p* < 0.0001) (Figure 1C,D). Aside from Omicron, the neutralizing capacities of convalescent sera varied depending on the variants. Sera with low or no neutralizing activity against one strain often showed a higher neutralizing activity against another strain (Figure 1A–C and 1E and Supplementary Figure S1A–C). Accordingly, there was a good but not perfect correlation between the neutralizing activities of convalescent sera against B.1 and Gamma (Spearman’s r = 0.6852, *p* < 0.0001) or Delta (Spearman’s r = 0.6924, *p* < 0.0001) (Figure 1E). The highest neutralizing activity against B.1 and VOCs was achieved by sera from patients with moderate disease (Figure 1F), thus highlighting the plasticity of the antibody response vis-à-vis different variants. The time elapsed between symptom onset and serum collection contributed only marginally and not significantly to the differences in neutralizing ability against B.1 (Supplementary Figure S1D).

#### 2.1.2. Serological Characterization of Convalescent Sera

**Figure 2:**
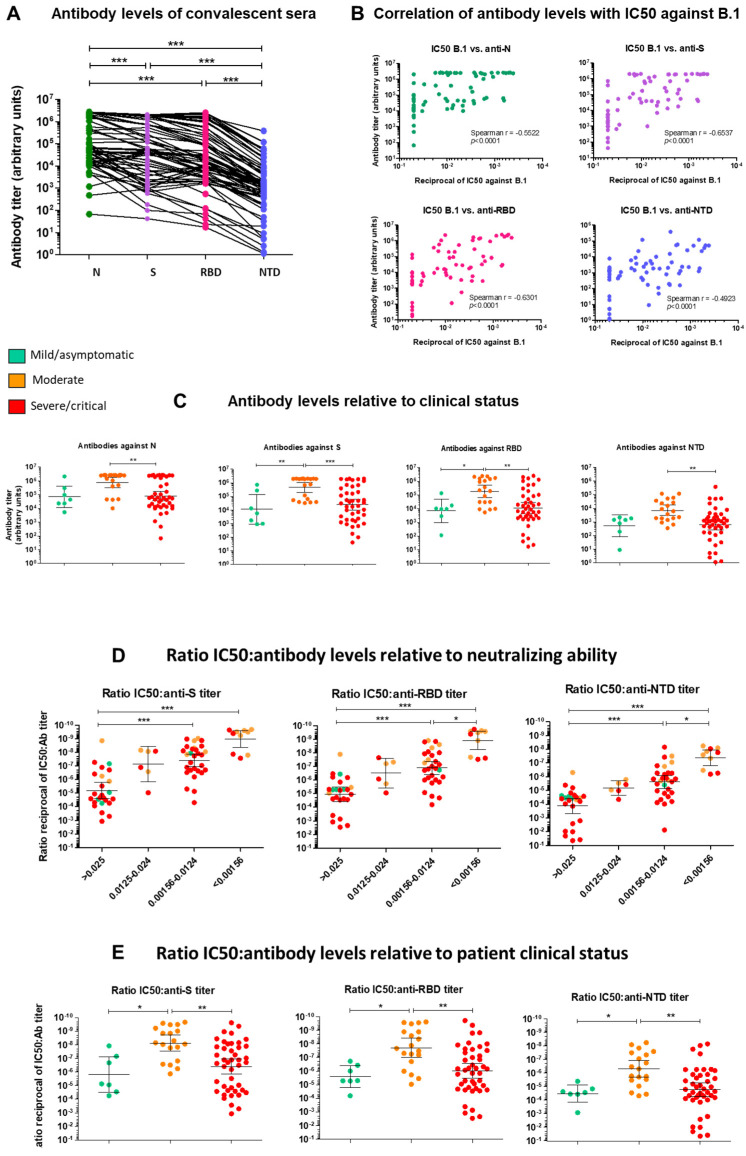
Serological characterization of convalescent sera. (**A**). Antibody levels in convalescent sera. Antibody levels against SARS-CoV-2 N (green), S (purple), the receptor-binding domain (RBD) (pink) or the N-terminal domain (NTD) (blue) of the Spike protein were measured in convalescent sera using the MSD V-plex platform for SARS-CoV-2. Antibody levels are reported as arbitrary units. (**B**). Correlation between half-maximal inhibitory concentration (IC50) of convalescent sera against B.1 (x-axis) and antibody levels against SARS-CoV-2 N, S, RBD and NTD (y-axis). The Spearman correlation coefficient (r) is indicated. (**C**). Antibody levels of convalescent sera stratified by disease severity. (**D**,**E**). Ratios of IC50 against B.1 to antibody levels against S, RBD and NTD stratified according to neutralizing activity (**D**) or to patient clinical status (**E**). For all analyses, differences between groups were compared using a Mann–Whitney U-test for comparison between two groups and a Kruskal–Wallis followed by a Dunn’s multiple comparison post-hoc test when three or more groups were compared. *p*-values < 0.05 were considered significant. *: *p* < 0.05; **: *p* < 0.01; ***: *p* < 0.001.

To gain insight into the target and efficacy of antibodies mediating the neutralizing activity, we measured antibodies targeting the viral Spike protein (S), the receptor-binding domain (RBD) and the S N-terminal domain (NTD) as well as antibodies targeting the viral Nucleocapsid (N), using the MSD V-plex platform. All patients had detectable antibodies against N, S and the RBD, ranging over five orders of magnitude (Figure 2A). Anti-NTD antibody levels were lower, and undetectable in a handful of patients with severe disease (Figure 2A). Antibody levels only moderately correlated with neutralizing ability (Spearman r = −0.5522 for N, Spearman r = −0.6537 for S, Spearman r = −0.6301 for RBD and Spearman r = −0.4923 for the NTD, *p* < 0.0001) (Figure 2B). In line with neutralizing ability, patients with moderate disease had significantly higher antibody levels against all viral determinants (N, S, RBD ant NTD) than patients with severe disease forms (Figure 2C), indicating a more rapid onset of targeted humoral response. 

We further assessed the neutralization activity relative to anti-S, anti-RBD and anti-NTD antibody levels (ratio IC50:antibody (Ab) levels). This ratio can be used as a proxy for antibody affinity as it takes into account the sum of individual neutralization abilities of antibodies [99]. As shown in Figure 2D, the IC50:Ab level ratio decreased markedly with increasing neutralizing ability for all antibodies (*p* < 0.001 for sera with IC50s < 0.0124 compared to non-neutralizing sera). Overall, the IC50:anti-S and IC50:anti-RBD ratios were similar, while the IC50:anti-NTD ratio was ~1 to 1.5 log_10_ higher, reflecting the fact that antibodies targeting the RBD represent the most abundant fraction of antibodies targeting S and account for most of the neutralizing ability. Again, the lowest ratio (highest neutralizing activity and highest Ab levels) was achieved by sera from patients with moderate disease (Figure 2E), suggesting high NAb levels mediate efficient neutralization.

### 2.2. Cross-Neutralization of Sera from Vaccinated Breakthrough Infection Cases

#### 2.2.1. Cross-Neutralization of BTI Sera

**Figure 3:**
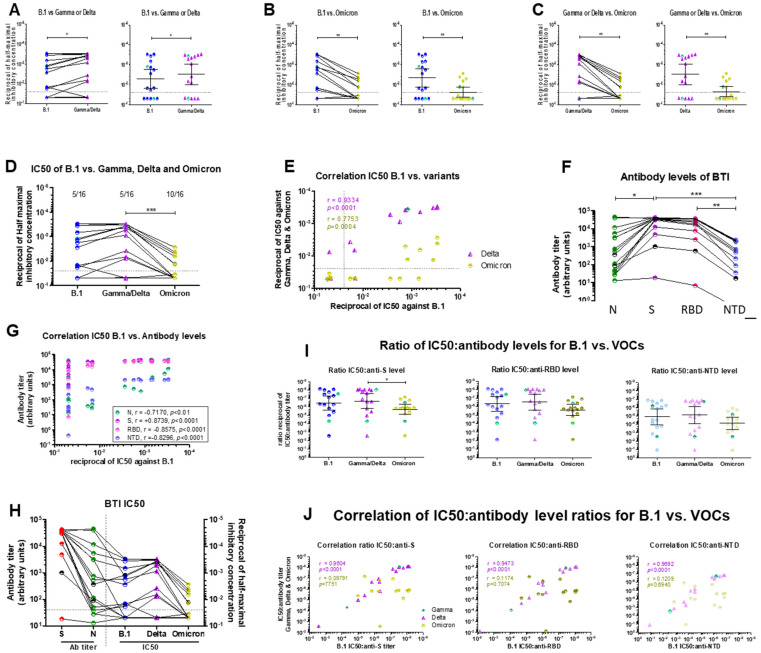
Neutralizing activity and serology of sera from vaccinated breakthrough infections (BTIs) against B.1, Gamma, Delta and Omicron BA.1. (**A**–**C**). Pairwise comparison of neutralization of B.1, Gamma, Delta and Omicron. Serial two-fold dilutions (starting 1:40, grey dotted line) of heat-inactivated sera from BTI cases collected around the time of infection were incubated for 1 h with 100 TCID50 of representative ancestral D614G (B.1), Gamma, Delta or Omicron BA.1 SARS-CoV-2 strains in viral growth medium. The infecting strain is indicated on the x-axis. Vero-E6 cells (10^4^ cells/ well) were infected with the mixture for 72 h. Virus-induced CPE was measured using the tetrazolium salt WST-8 as above. All infections were performed in triplicate wells. Percent survival was calculated relative to uninfected cells (100% survival) and the half-maximal inhibitory concentration for serum (IC50) was determined by inferring the 4-parameter nonlinear regression curve fit (GraphPad Prism v5). The top (100% survival) and bottom (no serum) values were unconstrained. IC50s for Gamma-BTI and Delta-BTI are grouped. Gamma-BTI sera are represented in green in each panel and were exposed to B.1, Gamma or Omicron BA.1. Delta-BTI sera are represented in blue, purple or gold for exposure to B.1 (blue), Delta (purple) or Omicron BA.1 (gold). (**D**). Comparison of IC50 for all strains. The infecting strain is indicated on the x-axis. The green symbols represent Gamma-BTI sera exposed to the indicated virus strain (B.1, Gamma or Omicron BA.1). The proportion of non-neutralizing sera is indicated above each data set. (**E**) Correlations between half-maximal inhibitory concentration (IC50) against B.1 (x-axis) and VOCs (y-axis) Gamma (green diamonds), Delta (purple triangles) and Omicron BA.1 (gold circles). The Spearman correlation coefficient (r) is indicated in purple for Gamma/Delta and in gold for Omicron in the panel. (**F**). Serology of BTI sera. Antibodies against SARS-CoV-2 N (Nucleocapsid), S (Spike), the Spike RBD or the Spike NTD were measured using the MSD V-Plex COVID-19 Coronavirus Panel 1 serology kit. Antibody levels are reported as arbitrary units. Sera from BTI cases infected with Gamma are in black; those infected with Delta are colored. (**G**). Correlation between IC50 against B.1 (x-axis) and antibody levels (y-axis) (green = anti-N, purple anti-S, pink = anti-RBD, blue = anti-NTD). The Spearman correlation coefficients (r) are indicated in the box. (**H**). Anti-S and anti-N antibody levels (left y-axis and left side of the panel) and half-maximal inhibitory concentrations (IC50) of BTI sera against B.1 (blue), Gamma/Delta (green or purple, respectively) and Omicron (gold) (right y-axis and right side of the panel). For IC50s, the infecting strain is indicated on the x-axis. Gamma BTI-sera are highlighted in black in all cases. (**I**). Ratios of IC50 against B.1 (blue circles), Gamma (green diamonds), Delta (purple triangles) and Omicron (gold circles) divided by antibody levels against S, RBD and NTD in BTI sera. The infecting strain is indicated on the x-axis. Gamma-BTI sera are always indicated in green and Delta-BTI sera are in blue for exposure to B.1, purple for Delta and gold for Omicron BA1. (**J**). Correlations between ratios of IC50:antibody (Ab) levels against S, RBD and NTD for BTI sera against B.1 (x-axis) and VOCs (y-axis). Color code: B.1: blue circles; Gamma: green diamonds, Delta: purple triangles, Omicron BA.1: gold circles. In all cases, green symbols represent Gamma-BTI exposed to B.1 (left panel), Gamma (middle panel) or Omicron BA.1 (right panel). For all analyses, differences between groups were compared using a Mann–Whitney U-test for comparison between two groups and a Kruskal–Wallis followed by a Dunn’s multiple comparison post-hoc test when three or more groups were compared. *p*-values < 0.05 were considered significant. *: *p* < 0.05; **: *p* < 0.01; ***: *p* < 0.001.

Next, we tested the neutralizing ability of sera from 16 vaccinated individuals infected with Gamma (2 cases) or Delta (14 cases) (breakthrough infection (BTI)) against the ancestral B.1 strain, the corresponding infecting VOC (Gamma or Delta) and Omicron BA.1. BTIs were infected between 15 July and 18 September 2021, when Gamma and Delta were the main circulating VOCs in Luxembourg. The median time elapsed since the second vaccine dose was 3.06 months (CI = 2.09–4.47). Most sera were collected at the time of diagnosis but time since symptom onset was not known. Typically, BTI sera could be categorized into two clearly distinct categories, non-neutralizing or very poorly neutralizing sera (eight sera) or highly neutralizing sera (eight sera) (Figure 3A). Among the non-neutralizing sera, five (four Delta, one Gamma) had no neutralizing ability against B.1 at the highest serum concentration tested (1:40). The geometric mean IC50 against the infecting VOC (Gamma for Gamma- BTI cases and Delta for Delta-BTI cases) was slightly but significantly lower (IC50 = 0.003038) than against B.1 (IC50 = 0.005131) (*p* = 0.058) (Figure 3A), indicating a better neutralizing ability against the infecting strain than against the wild-type virus the vaccines were designed on. The geometric mean IC50 could not be calculated for the two BTIs with Gamma alone, but they also fell into one of the two profiles, one having no neutralizing ability and one with good neutralizing ability (Figure 3A). In contrast to what was recorded for convalescent sera, the neutralizing profile was maintained across strains overall, such that all sera which neutralized B.1 at dilutions higher than 1:80 had similar or higher neutralizing ability against the corresponding VOC. Conversely, most sera which did not neutralize B.1 also failed to neutralize the infecting VOC (one Gamma and three of four non-neutralizing Delta-BTI sera) (Figure 3A). Therefore, while it is easily conceivable that individuals with no or low neutralizing ability would be susceptible to breakthrough infection, more than half the BTI cases had NAbs effective against Gamma or Delta. Furthermore, their sera cross-neutralized Delta better than the ancestral B.1 strain, arguing against the hypothesis that immune escape underlies the breakthrough infection in these cases. 

Next, we assessed the cross-neutralizing ability of these sera against Omicron BA.1. As shown in Figure 3B–D, all BTI sera featured a substantial drop in neutralizing ability against Omicron BA.1 (IC50 = 0.02358, *p* = 0.0105 compared to B.1 and *p* = 0.0067 compared to Delta). However, of the eight BTI sera with high neutralizing ability against B.1 and Gamma or Delta, six also retained partial neutralizing ability against Omicron BA.1 (Figure 3B–D). Accordingly, there was a very good correlation between the IC50s of BTI sera against B.1 and Gamma or Delta (Spearman r = 0.9334, *p* < 0001), and a lower correlation between B.1 and Omicron (Spearman *r* = 0.7753, *p* = 0.004) (Figure 3E).

#### 2.2.2. Serological Characterization of BTI Sera

All but one BTI serum had high antibody levels against S, including the RBD and the NTD (Figure 3F), in line with their vaccination status. In contrast, antibodies targeting N spanned a broad range (Figure 3F), suggesting that infection may have been going on for some days before diagnosis in some BTI patients. The neutralizing ability against B.1 correlated nicely with antibodies against S, the RBD and the NTD (Spearman r = −0.8739, *p* < 0.0001 for S, r = −0.8739, *p* < 0.0001 for RBD and r = −0.8575, *p* < 0.0001 for the NTD) and slightly less with antibodies against N (Spearman r = 0.7170, *p* < 0.01) (Figure 3G), confirming that breakthrough infection does not reflect a lack of circulating NAbs directed against the Spike protein. Higher anti-N antibodies were present in sera with higher neutralizing ability (Figure 3H). This observation coupled with the fact that patients with high antibody levels against S determinants did not necessarily have high anti-N antibody levels suggests either a different time elapsed since infection or a lag in the onset of the antibody response in some BTI cases. 

The IC50:Ab level ratio was lowest for S and the RBD and ~1.5 log_10_ higher for antibodies targeting the NTD, suggesting that most anti-S antibodies elicited by vaccines target the RBD, while antibodies targeting the NTD are much less abundant (Figure 3I). The IC50:Ab level ratio was similar for B.1 and Gamma/Delta for all antibodies targeting S (Figure 3J), reflecting the high cross-neutralizing ability of antibodies against the infecting virus. The IC50:Ab level ratio against Omicron was nearly 1 log_10_ higher (Figure 3F), again congruent with the lower efficacy of antibodies against this variant.

These figures highlight differences in the cross-neutralizing ability of convalescent sera in comparison to BTI sera. The former showed different cross-neutralization profiles against the Gamma and Delta VOCs and a retained noneutralizing activity against Omicron BA.1, while BTI sera showed more similarities across variants and only a partial loss in neutralization against Omicron BA. Furthermore, antibody levels in BTI sera correlated much better with neutralizing ability than antibody levels in convalescent sera.

### 2.3. Comparison of BTI and Convalescent Sera

**Figure 4:**
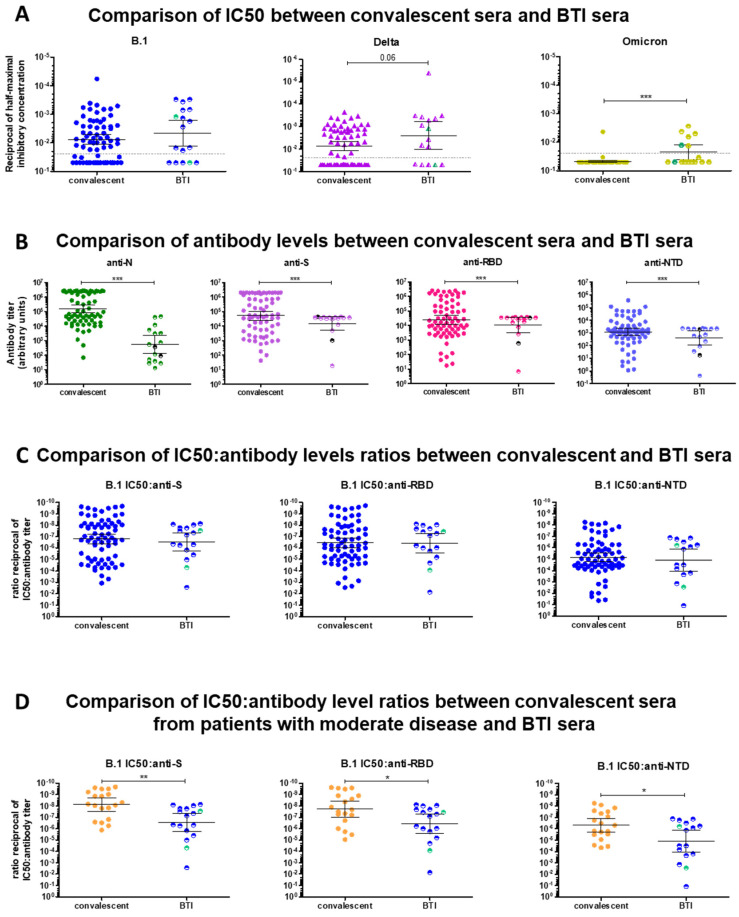
Comparison of neutralizing activities of BTI and convalescent sera against B.1, Gamma, Delta and Omicron. (**A**). Comparison of half-maximal inhibitory concentrations (IC50) of convalescent and BTI sera against B.1, Gamma/Delta and Omicron. The infecting strain is indicated above each panel. For BTI sera, the Gamma-BTI patients infected with Gamma are represented with green symbols and the Delta-BTI patients infected with Delta are represented with purple symbols. A pairwise comparison with Gamma was not possible since only two BTI infected with Gamma were available. The grey dotted line represents the 1:40 serum dilution cutoff. (**B**). Comparison of antibodies against N, S, RBD and NTD in BTI and convalescent sera. For BTI sera, the Gamma-BTI are represented with black circles and the Delta-BTI are represented in green for anti-N antibodies, purple for anti-S antibodies, pink for anti-RBD antibodies and blue for anti-NTD antibodies. (**C**). Comparison of the IC50:antibody (Ab) level ratios for BTI and convalescent sera. Cells were infected with B.1 and the IC50 of sera against B.1 is shown. For BTI sera, the Gamma-BTI are represented with green circles and the Delta BTI sera are represented with blue circles. (**D**). Comparison of the IC50:Ab level ratios for BTI and convalescent sera with moderate disease. Cells were infected with B.1 and the IC50 of sera against B.1 is shown. For BTI sera, the Gamma BTI sera are represented with green circles and the Delta BTI sera are represented with blue circles. For all analyses, differences between groups were compared using a Mann–Whitney U-test. *p*-values < 0.05 were considered significant. *: *p* < 0.05; **: *p* < 0.01; ***: *p* < 0.001.

A side-by-side comparison of convalescent and BTI sera showed that BTI sera had a higher neutralizing activity against B.1 as well as against VOCs, including Omicron BA.1 (Figure 4A). Six of the eight neutralizing BTI sera cross-neutralized Omicron, albeit less effectively than B.1 and the infecting VOC (Gamma or Delta). This result clearly indicates that a breakthrough infection with pre-Omicron VOCs induces an antibody response which cross-neutralizes Omicron BA.1, in contrast to convalescent sera. Remarkably, the higher neutralizing activity was achieved with significantly lower antibody levels against N, S, the RBD and the NTD (*p* < 0.0001 in all cases) in BTI sera compared to convalescent sera (Figure 4B), indicating that a higher neutralizing efficacy is reached with lower antibody titers, thus hinting at a greater affinity of antibodies. The neutralizing activity remained significantly higher (*p* < 0.001) when only patients with moderate infection, which displayed the highest neutralizing activity, were compared to BTI (not shown). Accordingly, the IC50:Ab level ratios were comparable for BTI and convalescent sera (Figure 4C and Supplementary Figure S2A), confirming that the lower IC50 (higher neutralizing ability) necessitated lower antibody titers. This observation held true for all VOCs and for anti-S, anti-RBD and anti-NTD antibodies. The convalescent sera from patients with moderate disease had higher neutralizing ability (Figure 1F) and higher antibody levels (Figure 2E) than the convalescent sera from patients with mild/asymptomatic disease or with severe or critical disease. We thus compared the IC50:Ab level ratio of convalescent sera from patients with moderate disease to the IC50:Ab level ratio of BTI sera. As shown in Figure 4D and in Supplementary Figure S2B, the IC50:Ab level ratio of patients with moderate disease was lower than that of BTI, indicating that higher neutralizing ability (lower IC50) necessitated higher antibody levels in unvaccinated patients than in BTI patients. Therefore, although antibodies elicited upon infection in vaccinated individuals are much less abundant than those elicited by infection in unvaccinated individuals, they appear to have a higher affinity than those elicited by infection alone. This held true in individuals with efficient neutralizing responses and contained disease manifestations (Figure 4D and Supplementary Figure S2B). This feature may either reflect a more targeted humoral response induced by vaccination over natural infection, or the superiority of hybrid immunity over immunity elicited by infection alone [36,93,100,101,102]. The observation that BTI patients with higher anti-N antibody levels had the highest neutralizing activity argues in favor of the second hypothesis.

## 3. Discussion

In this study we compared the neutralizing abilities and antibody levels of sera from unvaccinated individuals infected with the ancestral B.1 (D614G) strain (convalescent sera) and from vaccinated BTI individuals. Overall, ~30% of the convalescent sera and 50% of the BTI sera were non-neutralizing against B.1, Gamma and Delta (Figure 1A–D and Figure 3A–D). Convalescent and BTI sera which neutralized B.1 generally retained good neutralizing ability against Gamma and Delta, while they displayed a substantial decrease in neutralizing ability against Omicron BA.1 (Figure 1A–D and Figure 3A–D) in agreement with previous studies [24,25,26,27,28,45,46,47,48,56,57,68,72,73,74,75,76,77,78,81,95,99,103,104,105,106]. It is thought that the ability of Omicron BA.1 to elude NAbs reflects a combination of a higher affinity of the Spike protein for ACE2 [20,21,22], together with a TMPRSS-2-independent, endocytosis-mediated entry [20,107,108]. Furthermore, the Omicron lineage is phylogenetically and serologically distinct from the previous SARS-CoV-2 lineages. The Omicron Spike protein also adopts a distinct, more compact conformation and glycosylation patterns that shield it from NAbs [23]. The fact that prior infection and vaccination do not fully protect against infection with Omicron or symptomatic disease but do partly protect against severe disease [109] suggests that T-cells and memory B-cells [27,85,107], as well as other innate immune signatures [110] are involved. 

Although both convalescent and BTI sera were immunized against the ancestral pre-VOC strain, either through natural infection or through vaccination, we recorded qualitative differences within and between both groups of patients. First, the cross-neutralizing ability of convalescent sera was markedly strain/variant-dependent. Some sera failed to neutralize B.1 but readily neutralized Gamma or Delta or vice-versa (Figure 1A–D and Supplementary Figure S1A–C). Furthermore, there was a substantial heterogeneity in the neutralizing ability and antibody levels among unvaccinated convalescent patients. Patients with moderate disease generally had the highest antibody levels (Figure 2A–C) and the highest neutralizing activity against B.1 as well as against pre-Omicron VOCs (Figure 1F). The majority of convalescent sera which failed to neutralize B.1 were from patients with mild disease, where innate immunity sufficed to clear infection, or from patients with severe or critical disease (Figure 1F). The correlation and predictive power of IgG antibody levels with disease severity remains controversial. Some studies report that antibody levels and neutralizing activity parallel disease severity [111,112,113], while others find higher anti-Spike protein antibody levels in non-ICU patients and an association with survival [114,115]. Our results (Figure 2C,D) agree with the latter findings, and reinforce the importance of the potency and affinity of neutralizing antibodies in viral clearance and protection against severe disease. Accordingly, this distinction between groups of patients was sharper when the IC50:Ab level ratio was calculated (Figure 2E). This ratio was suggested to provide insight into the affinity of antibodies for the Spike protein [99,114,116] and thus highlights that the antibody response mounted by patients with severe disease is generally poorly neutralizing and may be “unfocused”. The IC50:Ab level ratio in sera from patients with moderate disease was lower than the IC50:Ab level ratio in sera from patients with severe or critical disease (Figure 2E) and in sera from BTI patients (Figure 4D), suggesting that unvaccinated patients required higher antibody levels to achieve similar neutralization. In contrast to convalescent sera, sera from BTI patients had a much more uniform cross-neutralizing pattern, as they were either non-neutralizing against all strains, or neutralizing against the ancestral B.1 strain and the infecting VOC (Figure 3A–D). Neutralizing activity of BTI sera correlated very well with antibody levels (Figure 3G), while this correlation was more modest for convalescent sera (Figure 2B), further highlighting qualitative differences in antibodies elicited by vaccines and by infection. Vaccination or hybrid immunity lead to a higher neutralizing ability and broader cross-neutralization spectrum (Figure 4A), despite lower overall antibody levels (Figure 4B,C), likely reflecting a higher antibody affinity. A direct comparison of convalescent sera and BTI sera is hampered by the fact that the time since breakthrough infection or symptom onset is not known for BTI patients. Thus, hints can only be inferred from high or low anti-N antibody levels. While memory B- and T-cells against Spike determinants are expected to rapidly react against Spike and produce NAbs, anti-N antibodies likely appear later, in a timeframe similar to unvaccinated individuals. It is thus likely the time since symptom onset in BTI cases with high anti-N levels and convalescent sera are comparable. Despite these uncertainties, although NAb titers may still rise in BTI patients with infection progression and resolution, higher antibody levels together with affinity maturation would probably translate in a higher neutralizing ability and further decrease the IC50:Ab level ratio. Longitudinal studies would be needed to confirm this hypothesis. Notably, most BTI patients had high antibody levels against S and the RBD and high neutralizing activity against B.1, Gamma and Delta, i.e., against their infecting VOC, indicating that breakthrough infection occurred despite the presence of NAbs. One possible explanation is that the increased affinity and faster binding of variant Spike proteins to ACE2 [4,20,21,22] may short-circuit NAbs in some patients. Alternatively, because we measured the presence of NAbs in serum, NAbs may be scarce at the site of infection (upper respiratory airways), allowing infection. These antibodies may however play a role in protecting against severe disease and death.

A second major difference between convalescent sera and BTI sera is that neutralizing BTI sera partially retained their neutralizing capacity against Omicron (Figure 3B–D), while only one convalescent serum was able to neutralize Omicron (Figure 1C,D). In our study, the proportion of BTI patients with neutralizing antibodies against Omicron BA.1 above the dilution cutoff of 1:40 was 37.5% (6/16, 5 Delta and 1 Gamma), which is less than the proportion reported by a previous study (~75%) [106]. The ability of Delta-elicited sera to cross-neutralize Omicron BA.1 is debated [48,49,68,80,91,92,93,94,117]. Our results clearly indicate that Gamma- or Delta-breakthrough sera with a high neutralizing activity against these VOCs are able to cross-neutralize Omicron BA.1, in agreement with previous studies [49,93,94,118], although our data only apply to vaccinated BTI infected with Delta and we cannot infer whether they extend to BTIs infected with other VOCs. Since breakthrough infection acts as a booster, this observation is congruent with the restoration of neutralizing ability induced by booster vaccination [27,28,44,46,48,57,80,81,82,83,84,85,86,87,88]. It is currently acknowledged that vaccination of recovered COVID-19 patients with mRNA vaccines (hybrid immunity) induces IgG titers similar to or higher than two doses of vaccines in SARS-CoV-2-naïve individuals [36,101,119] and protects better against Omicron BA.1 than vaccination alone [48,49]. Conversely, breakthrough infection after vaccination confers some cross-protection, while Omicron BA.1 infection alone offers limited protection against pre-Omicron and against Omicron BA.2 and BA.2-related sublineages [92,95,96]. Hybrid immunity appears to cross-protect more efficiently than infection or vaccination individually and seems to be more durable [36,49,100,101,102,120,121], likely as a result of antibody affinity maturation [67,99,122,123]. In our case, BTI patients were not followed longitudinally and the time elapsed since infection was not known. Nevertheless, the observation that patients with high anti-N antibody levels (>1000 arbitrary units) had a higher neutralizing ability (Figure 3H) suggests a longer time window since infection. Affinity maturation proceeds in infected patients in secondary lymphoid organs long after viral clearance from the upper respiratory airways [116,124,125,126,127,128], as well as after vaccination [67,94,99,106,122,129]. Further supporting the importance of ongoing affinity maturation, a breakthrough infection with Delta or Omicron after full vaccination with mRNA vaccines (2 doses), and more specifically the time window between the second vaccine dose and breakthrough infection, was reported to elicit cross-reactive antibodies effective against Omicron [49,93,99]. Although we could not confirm the role of time between vaccination and breakthrough in this study, it is possible that the time elapsed since vaccination (~3 months) and the combination of different strains (Wuhan-based vaccines + infection with Gamma or Delta) may play a role. One recent report comparing the neutralization of Omicron sublineages BA.1, BA.2, BA.4 and BA.5 by sera from infected-vaccinated and BTI individuals further shows that sera from BTI are much more neutralizing than sera from infected-vaccinated individuals [19]. This observation probably reflects the combination of new antibody responses neutralizing different strains together with affinity maturation of existing NAbs in BTI sera, while in infected-vaccinated sera, affinity maturation is “constrained” towards the ancestral strain. It is however noteworthy that the BTI patients in the aforementioned study were infected with Omicron, while the BTI cases of our study were infected with pre-Omicron, phylogenetically more distant strains.

It is difficult to infer the relative contributions of different immunization means (mRNA vaccines, DNA vector vaccines, replicating virus) on one hand and phylogenetically and serologically distinct determinants on the other hand to the qualitative superiority of hybrid immunity over infection or vaccination alone. The observation that Omicron BA.1 infection alone [92] and vaccines based on Omicron BA.1 only [117] trigger neutralizing responses restricted to Omicron BA.1 suggest that viral variability plays a key role. The fact that booster strategies based on pre-Omicron VOCs Beta or Delta did not neutralize Omicron BA.1 better than the wild-type booster [80] lends further support to this view. In contrast, heterologous Omicron BA.1-based booster vaccines [130] and bivalent booster vaccines based on wild-type + Omicron BA.1, Delta + Omicron BA.1 or hybrid Omicron BA.1/Delta determinants induce higher neutralizing humoral responses and T-cell responses against all Omicron sublineages as well as against pre-Omicron VOCs than homologous (wild-type) or pre-Omicron (Beta or Delta) booster vaccines in humans and mice [80,117,131,132,133]. Our results also indirectly confirm the serologic similarities between B.1 and Delta (Figure 1B,D and Figure 3A,D,E) on one hand and the serotypic specificity of the Omicron lineage and sublineages on the other. Given the high number of mutations harbored by Omicron lineages, and their overlap with pre-Omicron VOCs (e.g., Spike protein positions 69-70, 417, 484, 452, 501, 681), it is easily conceivable that heterologous vaccination or bivalent vaccines shape immune responses towards a higher neutralization efficacy through a broader range of epitopes and funnel affinity maturation towards a spectrum of mutated epitopes. Besides updating vaccines to best counter new lineages and serotypes as they emerge, combining vaccine types may further help fuel antibody levels, given that heterologous vaccination combining adenovector-based and mRNA-based vaccines or boost enhances NAb levels and durability [57,85], reminiscent of immunity conferred by hybrid immunity. The time window between boosters may further improve neutralizing antibody rebounds, either seasonally as is recommended for the Influenza Virus, or perhaps based on more personalized schemes upon NAb measures.

One shortcoming of this study is that it only includes a small number of BTI cases, particularly infected with Gamma. This reflected the relatively small number of breakthrough infections that required hospital admission. Despite these small figures, our results highlighted discrete patterns among BTI and significant differences between unvaccinated-infected and BTI humoral responses. Another caveat is that we could not compare the immune responses from unvaccinated-individuals and breakthrough infections infected with the same variant. Again, this situation ensued from the epidemic dynamic landscape, since vaccines were not available at the time the B.1 strain was circulating while VOCs had supplanted the ancestral strain after the roll-out of the vaccine campaigns. Nevertheless, since most vaccines were designed on the ancestral Wuhan stain, the epitopes targeted by antibodies elicited by infection and by vaccination should be comparable. Furthermore, the epidemic landscape on which the Omicron wave emerged is made of a multiplicity of past infections, different vaccines, vaccination times and patterns. As current vaccines are designed on the ancestral Wuhan strain and most BTI cases are due to Delta or Omicron, the data we report here are still representative of the grounds on which Omicron emerged. Moreover, while most individuals in Europe and the US have been vaccinated, in developing countries a large proportion of the population has not been vaccinated. In such contexts, prior immunity is only due to prior infection. Until now, VOCs have emerged in a context of suboptimal neutralization. As increasing numbers of individuals become infected/reinfected or receive different vaccines and (heterologous) boosters, SARS-CoV-2 variants will have to continue their evolutionary adaptation to overcome pre-existing immunity elicited by vaccines and by infections with distinct variants. It is tempting to speculate that future variants will emerge from a completely distinct lineage, generating one or more serotypes [134]. Only those variants with transmissibility rates higher than Omicron and with the ability to evade prior immunity will be able to supplant the current Omicron subtypes and recombinants. A close and rapid monitoring will be essential to assess their pathogenic potential and their susceptibility to NAbs elicited by previous VOCs. As Omicron variants are the main culprits for current BTI cases and elicit broad NAb responses [36], their cross-neutralizing ability will likely shape viral evolution. It will thus be essential to monitor the cross-neutralizing ability of Omicron-infected sera against emerging VOCs.

In conclusion, our data confirm and extend previous reports on the full immune escape of Omicron BA.1 against neutralizing antibodies elicited by prior infection. They further underline the protective role of hybrid immunity and the better cross-neutralizing ability conferred by infection in vaccinated individuals compared to unvaccinated individuals. Moreover, the overlap of breakthrough infections due to Omicron sublineages has focused most research efforts on the cross-neutralization within Omicron sublineages. Our data on the cross-neutralization of antibodies induced by infection with pre-Omicron variants against Omicron BA.1 shed light on the epidemic landscape on which Omicron emerged and spread. As new SARS-CoV-2 lineages with different serotypes may still emerge, a better understanding of the dynamics of humoral responses will help appreciate the impact on the epidemic. 

## 4. Materials and Methods

### 4.1. Patient Samples

Serum from 70 unvaccinated patients (hereafter “convalescent sera”) who were infected between March and July 2020 and serum from 16 vaccinated patients with breakthrough infection (2 Gamma and 14 Delta) (hereafter “BTI sera”) who were infected between 15 July and 20 September 2021 were analyzed. All patients had RT-PCR-confirmed SARS-CoV-2 infection. The study was approved by the LIH Institutional Review Board (study number 14718697-NeutraCoV). Anonymized patient left-over samples collected at the Centre Hospitalier de Luxembourg (CHL) were used for the setup of serological and virological tests in agreement with GDPR guidelines. No clinical data were available other than the time since symptom onset and the degree of disease severity recorded by the clinician for the unvaccinated patients. Disease severity stratification was as follows: *Mild/asymptomatic* patients (8 patients) presented flu-like symptoms or no symptoms; patients with *Moderate* disease (19 patients) had fever, flu-like symptoms, anosmia, fatigue, gastrointestinal disturbances, but did not require hospitalization or oxygen supplementation; patients with *severe* or *critical* disease (43 patients) were admitted to the hospital, required oxygen supplementation and/or intensive care. For BTI cases, data on the lineage of the infecting strain and the time since 2nd vaccine dose were provided by CHL. Convalescent sera were collected during acute infection (median 14 days, IQR 9-20), while the BTI sera were collected at the time of diagnosis but time since symptom onset was not available. 

### 4.2. Cells

Vero-E6 cells (a kind gift from Dr. Thorsten Wolff, Influenza und Respiratorische Viren, Robert Koch Institute, Germany) were maintained in DMEM supplemented with 10% fetal bovine serum (FBS), 2 mM L-glutamine, 50 µg/mL penicillin and 50 µg/mL streptomycin (all from Invitrogen, Merelbeke, Belgium). For infection experiments, 2% FBS was used (hereafter referred to as viral growth medium, VGM).

### 4.3. Serology

The MesoScale Diagnostics (MSD) V-Plex COVID-19 Coronavirus Panel 1 serology kit (K15362U) was used according to the manufacturer’s recommendations to determine the IgG profile of sera (MesoScale Diagnostics, Rockville, MD, USA). This multiplex assay includes SARS-CoV-2 antigens (N, S, RBD, NTD) as well as Spike proteins from other Coronaviruses (SARS-CoV, MERS-CoV, OC43, HKU1) and Influenza A Hemagglutinin H3. 

### 4.4. Virus Isolation and Titration

SARS-CoV-2 strains D614G and VOCs (Gamma, Delta and Omicron BA.1) were isolated from anonymized left-over patient nasopharyngeal swabs (NPS) collected from patients at the CHL. For isolation, 500 µL of residual swab preservation medium was added to Vero-E6 cells (1.2 × 10^6^ cells) in VGM and cytopathic effect (CPE) was monitored visually daily. Viral supernatant was used to constitute a viral stock by infecting Vero-E6 cells in a second passage. The viral supernatant from passage 2 was centrifuged and stored at −80 °C until use. All experiments were performed with the same viral stock. Viral strains present in the original material (swabs) were identified through next-generation sequencing and the Spike protein was resequenced after the second passage to verify sequence conservation. We isolated representative strains for B.1 (D614G, pre-VOC), Gamma, Delta and Omicron (sublineage BA.1). 

The 50% tissue culture infectious dose (TCID50) was assessed by titrating viral strains on Vero-E6 cells in sextuplicate wells. Briefly, 10^4^ cells/well in a 96-well microtiter plate were infected with 200 µL of serial 10-fold dilutions of isolated virus (starting at 1:10 in VGM) for 72 h at 37 °C with 5% CO_2_. Virus-induced CPE was measured using the tetrazolium salt WST-8, which is cleaved to a soluble strongly pigmented formazan product by metabolically active cells (CCK-8 kit, Tebu-Bio, Antwerp, Belgium). After 72 h of infection, 10 µL of CCK-8 solution was added to culture wells and incubated for 3 h at 37 °C. Optical density at 570 nm was then measured. Virus-exposed wells were compared to uninfected wells (100% survival). The threshold for infection was set at 75% cell survival (i.e., all virus-exposed wells with <75% viable cells were considered infected) based on preliminary comparative experiments with visually recorded CPE and crystal violet staining. The TCID50 was calculated according to the Reed and Muench method.

### 4.5. Live-Virus Neutralization Assay

Serial two-fold dilutions of heat-inactivated (30 min at 56 °C) patient serum were incubated 1 h with 100 TCID50 of virus in VGM. The mixture (200 µL/well) was then inoculated on Vero-E6 cells (10^4^ cells/ well in a 96-well microtiter plate) and cells were cultured for another 72 h at 37 °C with 5% CO_2_. A positive control (no serum) and an uninfected control (no serum-no virus) were included in each plate to assess maximum infection (no serum) and minimum (no virus) values. All infections were performed in triplicate wells. Virus-induced CPE was measured using the tetrazolium salt WST-8 as above. Percent survival was calculated relative to uninfected cells and the half-maximal inhibitory concentration for serum (IC50) was determined by inferring the 4-parameter nonlinear regression curve fit (GraphPad Prism v5). The top and bottom values were unconstrained. The neutralizing capacity of sera was assessed against B.1 (D614G strain), Gamma, Delta and Omicron BA.1 for convalescent sera, and against B.1, Omicron BA.1 and the variant that caused breakthrough for BTI (i.e., Gamma-BTI was evaluated against Gamma and Delta-BTI against Delta). To ensure equivalent infection levels, a “back-titration” was performed in each experiment with each of the viral strains. Briefly, the viral dilution used to infect cells in the presence or absence of serum dilutions was titrated as above, in 10-fold dilutions in VGM and the TCID50 was calculated using the Reed and Muench formula to verify that the virus inoculum was 100 TCID50. 

### 4.6. Statistical Analyses

Statistical analyses were performed using GraphPad Prism v5. The Shapiro–Wilk test was used to verify distributions. Differences between groups were compared using a Mann–Whitney U test for comparison between two groups and a Kruskal–Wallis signed-rank test followed by a Dunn’s post-hoc test for multiple comparisons since most datasets were non-normally distributed. Correlation coefficients (r) were determined using a Spearman’s rank correlation. *p*-values < 0.05 were considered significant.

## Supplementary Materials

The following supporting information can be downloaded at: https://www.mdpi.com/article/10.3390/ijms23147675/s1.
