# Supplementary material for: Pre-Omicron Vaccine Breakthrough Infection Induces Superior Cross-Neutralization against SARS-CoV-2 Omicron BA.1 Compared to Infection Alone
Source: Int J Mol Sci. 2022 Jul 12;23(14):7675. doi: 10.3390/ijms23147675 (PMC9320437; doi:10.3390/ijms23147675)
Supplement: Supplementary file 1 [file ijms-23-07675-s001.zip › ijms-1799791-supplementary.pdf]

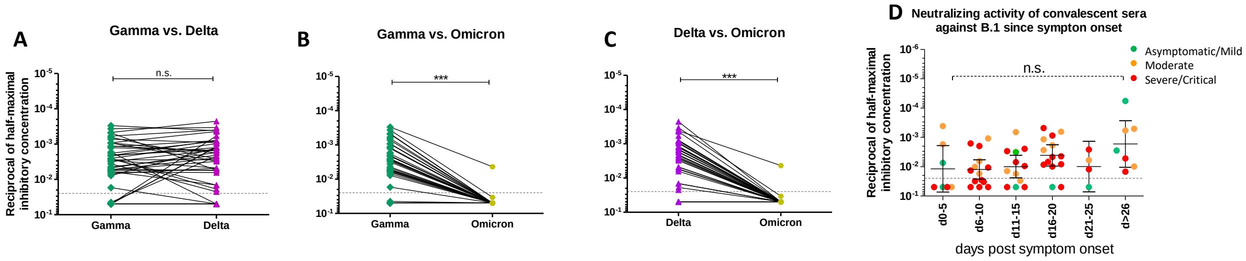

Supplementary Figure S1. A-C. **Pairwise comparison of half-maximal inhibitory concentrations (IC50) of convalescent sera against VOCs.** Half-maximal inhibitory concentrations against each VOC were compared. Cells were infected with the strain indicated on the x-axis. **D.** Neutralizing ability of sera against B.1. in relation to time since symptom onset for patients with mild/asymptomatic disease (green), moderate disease (orange) or severe/critical disease (red). The grey dotted line represents the 1:40 serum dilution cut-off. Differences between groups were compared using Mann-Whitney test (A-C) or a Kruskal-Wallis followed by a Dunn's multiple comparison post-hoc test (D). P-values < 0.05 were considered significant. *: p<0.05; **: p<0.01; ***: p<0.001

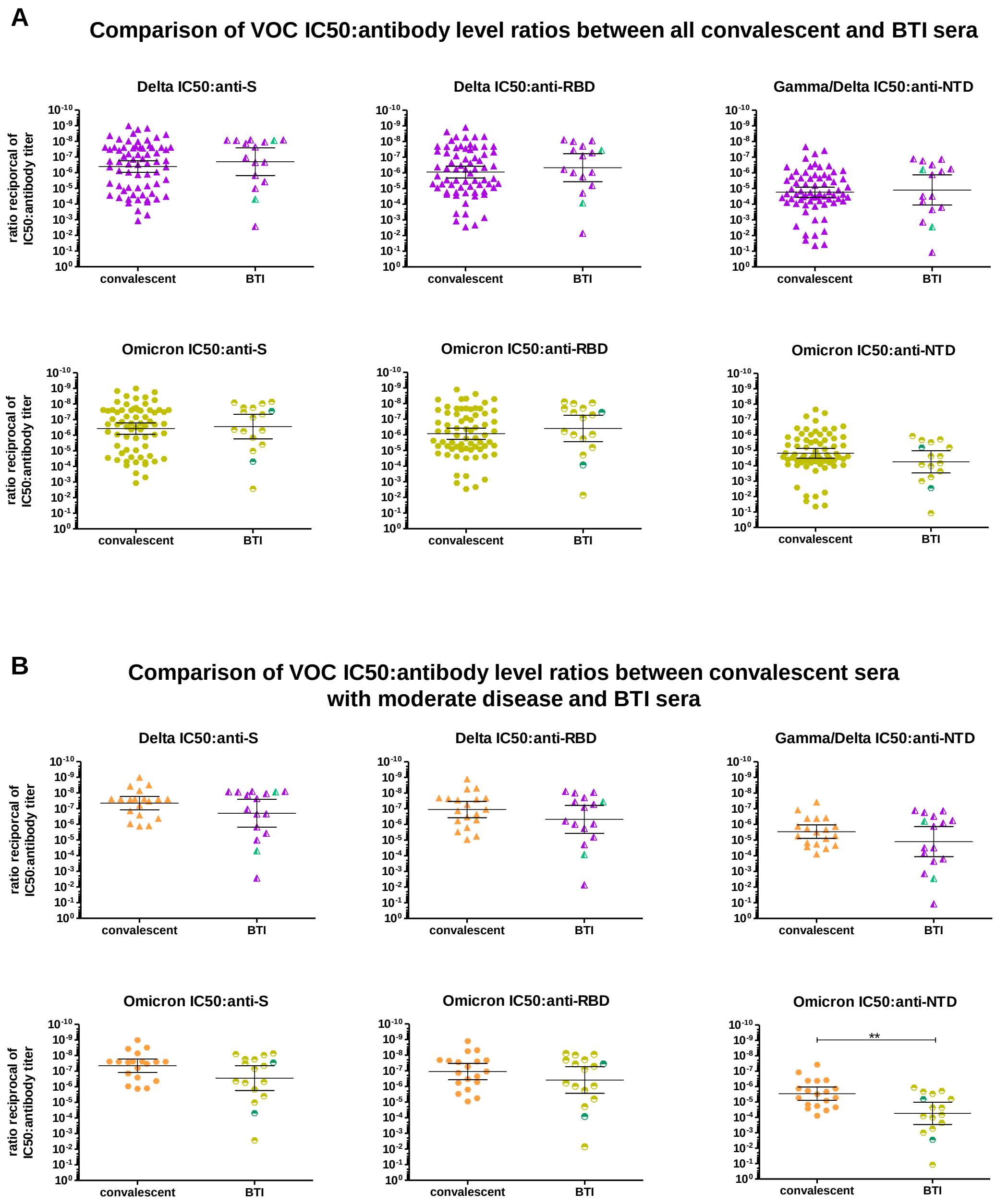

Supplementary Figure S2. Comparison of the Delta (purple) and Omicron (gold) IC50:antibody (Ab) level ratios for convalescent and BTI sera for all convalescent patients (A) and for convalescent patients with moderate disease (B). The infecting strain is indicated above each panel. For BTI sera, the Gamma-BTI sera are represented with green circles. Differences between groups were compared using Mann-Whitney test. P-values < 0.05 were considered significant. *: $p<0.05$; **: $p<0.01$; ***: $p<0.001$.
